# Linking socio-sexual and vocal behaviour with faecal progesterone and oestrogen metabolite levels in Southern white rhinoceros females

**DOI:** 10.1093/conphys/coab098

**Published:** 2021-12-29

**Authors:** Julia Jenikejew, Jella Wauters, Martin Dehnhard, Marina Scheumann

**Affiliations:** 1 Institute of Zoology, University of Veterinary Medicine Hannover, Bünteweg 17, 30559 Hannover, Germany; 2 Department of Reproduction Biology, Leibniz Institute for Zoo and Wildlife Research, Alfred-Kowalke-Straße 17, 10315 Berlin, Germany

**Keywords:** zoo, reproduction monitoring, olfactory behaviour, oestrus cycle, faecal steroids, Ceratotherium simum

## Abstract

Progesterone and oestrogen are the main gonadal steroid hormones that regulate the ovarian activity and induce the fertile oestrus period in females. The monitoring of this receptive phase is particularly decisive for captive breeding and is commonly based on the observation of female behavioural patterns that coincide with their hormonal oestrus. However, in the white rhinoceros (WR), a species that is well known for its impaired reproductive rate in captivity, the female behavioural and vocal indicators of receptivity have not been systematically investigated or linked to their hormonal states so far. In order to close this gap, we combined behavioural and acoustic recordings, conducted over an average period of 32 days, with the analysis of faecal progesterone and oestrogen metabolite concentrations (fPM and fEM) in 27 adult Southern white rhinoceros (*Ceratotherium simum simum*; SWR) females from 10 European zoos. For eight of the study females, we were able to detect a receptive period indicated by their acceptance of sexual behaviour from the bulls. The comparison of behaviour and vocalization between receptive and non-receptive periods in these females demonstrated that particularly presenting and marking behaviour distinctly peaked during the receptive period, indicating the significance of olfactory signalling for female reproductive behaviour. Based on the analysis of fPM profiles, we were able to identify different reproductive states (cycling, non-cycling, pregnant) in 21 of 27 study females. In contrast, fEM profiles proved to be unsuitable for the detection of ovarian activity. For the majority (five of six females for which behavioural receptivity and endocrine cyclicity were determined), a coincidence of their receptive period and the hormonal oestrus, indicated by a nadir in fPM levels, could be detected. Conclusively, this study revealed a comprehensive behavioural repertoire that reflects the hormonal oestrus in SWR females and can therefore be reliably used for non-invasive ex situ reproduction monitoring.

## Teaser

Behaviour, vocalization and faecal metabolite concentrations of progesterone (fPM) and oestrogen were compared between receptive and non-receptive periods in female white rhinoceroses. Affiliative and olfactory-related behaviour peaked during receptive period, coinciding with hormonal oestrus and nadirs in fPM concentrations. The results reveal a sexual behavioural repertoire reflecting the hormonal state.

## Introduction

Sexual behaviour, encompassing all behavioural patterns that lead up to and involve mating, is required for reproduction and hence for the survival of the species. In females, sexual behaviour mainly comprises the components with which they initiate copulation and respond to sexual initiations of the mating partner (Nelson, 2011). These behavioural patterns are largely universal among mammals and include the seeking of vicinity of males, adopting species-specific coital postures or gestures as well as initiating body contact to the male (Beach, 1976). In most, but not all, mammalian species sexual behaviour is restricted to the oestrus phase, as it signals the female’s receptivity to the male, enabling mating only when the female is on her peak of fertility (Jennings and de Lecea, 2020; Wallen and Zehr, 2004). Outside of this period, females usually reject males.

In mammals, sexual behaviour in both sexes is largely regulated by gonadal steroid hormones. While in males the main steroid hormone is testosterone, in females the ovarian steroids progesterone and oestrogen are known to modulate the endocrine cycle (Jennings and de Lecea, 2020). Starting with puberty, sexually mature females experience regular cycles of alternating rises of oestrogen and progesterone levels as a response of the interplay of hormones produced by the hypothalamus, pituitary and ovaries (Hobeika *et al.*, 2020; Young and McNeilly, 2010). Generally, the oestrous cycle is subdivided into the follicular and the luteal phase (Hayssen and Orr, 2017; Nelson, 2011). During the follicular phase oestrogen levels rise in parallel to the growing follicles, eventually stimulating the secretion of follicle-stimulating hormone and mainly luteinizing hormone (LH), resulting in the so-called LH surge (Hobeika *et al.*, 2020; Young and McNeilly, 2010). Shortly thereafter, the oocyte is released from the dominant *graafian* follicle into the fallopian tube, a process known as ovulation, marking the most fertile phase of the cycle (Brown, 2018; Hayssen and Orr, 2017). With the subsequent onset of the luteal phase, the ovulated follicle is transformed into the *corpus luteum*, which begins to secrete high levels of progesterone, preparing the oestrogen-primed endometrium for potential implantation (Brown, 2018; Nelson, 2011). In case of successful fertilization, the progesterone-dominated phase is prolonged throughout the gestation period until shortly before parturition. In case pregnancy does not occur, the *corpus luteum* regresses by the end of the luteal phase, inducing a new cycle (Brown, 2018; Nelson, 2011).

For a wide variety of different mammalian taxa, the proportion of female reproductive hormones during different oestrous phases has been directly linked to the occurrence of sexual behaviour. For instance, female gorals display sexual behaviour, most notably lifting up the tail, during the time of elevated oestrogen levels (Khonmee *et al.*, 2014). Also, in goats, female-specific oestrus behaviour such as decreased appetite, restlessness, increase in vocal activity, frequent urination and accepting mounting behaviour of males was clearly associated with peaking oestrogen levels (SankarGanesh *et al.*, 2014). Similarly, increased vocalization and rolling correlated positively with oestrogen levels in Asiatic lionesses (Umapathy *et al.*, 2007), while in clouded leopard females increased oestrogen concentrations were associated with behavioural oestrus, entailing decreased food intake, lordosis posture and increased affiliative behaviour (Brown *et al.*, 1995). Also, in primates, the most fertile phase has been correlated with distinct display of sexual behaviour, peaks in oestrogen as well as a nadir in progesterone levels (Engelhardt *et al.*, 2005).

The concurrence between behavioural receptivity and hormonal oestrus is of particular interest for ex situ breeding, as it entails crucial information on the most reasonable time point for mate pairing (Graham, 2004; Lindburg and Fitch-Snyder, 1994). Especially in solitary species, staging of mating can only occur when it can be ensured that the female will accept the sexual interactions of the male, which can only be specified by clear behavioural indicators. For instance, in female koalas the convulsive hiccoughing behaviour called ‘jerking’ as well as bellowing are the most conspicuous indicators for oestrus (Johnston *et al.*, 2000), while in cheetahs male sexual arousal (penile erections and stutter-barking) as a reaction to olfactory cues of oestrous females has been successfully implemented as a signal for pairings (Lindburg *et al.*, 1993). However, in order to correctly interpret the behavioural indicators of receptivity, it is necessary to not only know the entire behavioural repertoire but also be able to align it with the hormonal states of the females (Whitham and Wielebnowski, 2013). For instance, in giant pandas increased scent marking, lordosis posture and bleat vocalizations are well-established indicators for peaking oestrogen values, which are followed by a 48-hour fertility window that can be considered for mate pairing (Kersey *et al.*, 2016).

While endocrine measurements present clear physiological parameters, they can only be consulted retrospectively, whereas behavioural and vocal indicators can be identified and evaluated immediately. A successful integration of these approaches can provide evidence of how hormonal states might be reflected in behaviour and vocalization, and thus help the monitoring of the reproductive state and animal management decisions on a day-to-day basis, especially in species that rely heavily on ex situ conservation. However, to date the relationship between behavioural markers and hormone profiles is still not fully understood in the majority of mammalian species (Ganswindt *et al.*, 2012).

In this respect, the white rhinoceros (*Ceratotherium simum*, WR) is a special case, as particularly the female’s role in the reproduction of this megaherbivore species has received major attention in recent years, with studies mainly addressing the issue of the deficient reproduction rate in captivity (e.g. Hermes *et al.*, 2004; Schwarzenberger *et al.*, 1998; Swaisgood *et al.*, 2006). There have been many endocrinological investigations that repeatedly pinpointed the problem to aberrant ovarian activity in the majority of the females of reproductive age (reviewed by Roth *et al.*, 2018), partly attributing it to unnaturally long nulliparous phases that are observed in this species and the subsequent pathologies that come with that (Hermes *et al.*, 2004,^2006^). However, so far these studies were carried out without correlating the endocrine investigations with systematic behavioural analyses.

Commonly, the display of male courtship (attempts of mounting and copulation) has been used as behavioural indicator in previous studies, as it is known to coincide with the female oestrus (Brown *et al.*, 2001; Patton *et al.*, 1999; Swaisgood *et al.*, 2006). Moreover, Radcliffe *et al.* (1997) also included accepting male sexual behaviour as well as urine squirting and lifting up the tail as typical oestrus behaviour when evaluating the reproduction of WR females. Nonetheless, these behavioural indicators of receptivity have never been empirically proven and it has not been examined if there might be other indicators as well as how they develop throughout the female oestrous cycle or what might affect them.

In the wild, WR males are known to play the active role and initiate courtship and mating by approaching cycling females and guarding them for several weeks during their most fertile phase (Owen-Smith, 1975; Penny, 1987). A recent study clearly demonstrated that, also in captivity, WR males approach and follow the female during her receptive period, while distinctly displaying their sexual motivation by uttering the contact call Pant (Jenikejew *et al.*, 2021). This study also showed that during the receptive period, males spend an increased amount of time close to the female and display higher rates of affiliative behaviour before eventually mounting (Jenikejew *et al.*, 2021). So far, however, female sexual behaviour in WR has been studied less extensively. While it is well established that they only tolerate males’ sexual interactions during their receptive phase (Owen-Smith, 1975; Owen-Smith, 1988), it is not known if they also use the Pant call in the socio-sexual context or if they prefer other communication modalities to signal their receptivity. To close this gap, in the present study we aimed to provide a complementary approach to the previous investigations in male WR and gather empirical information on the sexual behaviour of female WR.

In order to do so, we integrated the different approaches of behavioural and vocal analyses as well as endocrinology in order to create a comprehensive picture of reproduction in WR females, thereby providing a foundation for an accessible non-invasive monitoring tool in captivity. We investigated the development of female behavioural and vocal parameters during the period when they accepted sexual interactions from males to emphasize potential indicators that would distinctly peak during the receptive period. Subsequently, we analysed fPM and fEM levels to determine the ovarian cycle. Finally, yet importantly, we examined if the hormonal oestrus of cyclic females temporally coincided with their receptive period and if the baseline fPM levels were affected by the occurrence of a cycle or the male state.

## Methods

### Study sites and animals

Overall, the data were collected from 27 adult female Southern white rhinoceros (*Ceratotherium simum simum*; SWR) at 10 European zoological institutions. Study females were kept in either one of five group categories varying in size and composition, consisting of the following: (1) one adult bull, at least two adult females and at least one juvenile; (2) one adult bull and at least two adult females; (3) one adult bull and one adult female; (4) three adult females and at least one juvenile; or (5) four adult females, two subadults and three juveniles (Table 1). Correspondingly, study females that were housed in direct contact with an adult bull (categories 1–3) were further classified according to whether or not they accepted sexual behaviour from the bull during the observation period (Table 1).

**Table 1:** Information on study females during observation period

ID	Age (years)	Sampling year	Zoo	Group category	Offspring (age in months)	Accepted sexual interactions from bull	Sampled period (days)	Number of daily samples	Sufficient sample rate	Hormonal cycle
Amelie	7	2014	Osnabrück	2		Yes	31	29	Yes	No
Marsita	9	2014	Osnabrück	2		Yes	29	10	No	NA
Lia	12	2014	Osnabrück	2		No	30	10	No	NA
Chris	9	2014	Augsburg	2		Yes	56	53	Yes	Yes
Kibibi	9	2014	Augsburg	2		Yes	56	53	Yes	Yes
Baby	43	2014	Augsburg	2		No	56	47	Yes	Yes
Shakina	9	2014	Dortmund	4	5		28	26	Yes	No
Jasira^a^	9	2014	Dortmund	4			26	24	Yes	No
Natala	30	2014	Dortmund	4			28	25	Yes	No
Temba	17	2015	Erfurt	2		No	40	18	Yes	No
Numbi	18	2015	Erfurt	2		No	40	18	Yes	No
Uzuri	10	2015	Hodenhagen	1	25	No	32	8	No	NA
Kianga	11	2015	Hodenhagen	1	19	No	30	9	No	NA
Claudia	17	2015	Hodenhagen	1	20	Yes	34	7	No	NA
Doris	43	2015	Hodenhagen	5			38	16	Yes	No
Cera	21	2015	Gelsenkirchen	2		No	25	15	Yes	No
Tamu	23	2015	Gelsenkirchen	2		No	25	15	Yes	No
Clara	12	2018	Schwerin	2		Yes	27	12	Yes	Yes
Karen	15	2018	Schwerin	2		Yes	29	18	Yes	Yes
Jane	19	2018	Münster	1	14	Yes	31	24	Yes	Yes
Vicky	32	2018	Münster	1		No	25	8	No	NA
Yoruba	11	2018	Amnéville	4	26		31	22	Yes	No
Hekaw	14	2018	Amnéville	4	8		31	22	Yes	No
Lucy^a^	16	2018	Amnéville	3		No	31	22	Yes	No
Tala	19	2018	Amnéville	4	18		31	21	Yes	Yes
Jamala	5	2019	Knuthenborg	2		No	32	31	Yes	No
Bodil	22	2019	Knuthenborg	2		No	32	31	Yes	No

^a^Study female was pregnant during sampling period.

Group categories: 1—one adult bull, at least two adult females and at least one juvenile; 2—one adult bull and at least two adult females; 3—one adult bull and one adult female; 4—three adult females and at least one juvenile; 5—four adult females, two subadults and three juveniles.

Detection of hormonal cycle not possible when study females were sampled on <3 days/week (NA).

### Vocal and behavioural data collection

Over an average period of 32 days, simultaneous acoustic and behavioural recordings were taken of all individuals in the groups using focal animal sampling (Altmann, 1974). Each focal animal was observed for 10 minutes per session, resulting in 20–40 minutes daily observation time randomly distributed between 8 am and 6 pm.

Video recordings were made using a digital camcorder (Sony DCR-SR36E, Sony Corporation, Tokyo, Japan). Audio recordings were made using a Sennheiser omni-directional microphone (Sennheiser MKH 8020, Sennheiser electronic GmbH & Co. KG, Wedemark-Wennebostel, Germany; flat frequency response from 10 to 20 000 Hz ± 5 db) that was equipped with a wind shield and a boom pole. The microphone was connected to a digital recording device (Sound devices 702T State Recorder, Sound Devices LLC, Reedsburg, USA; frequency response: 10–40 000 Hz; settings: 44.1 kHz sampling rate, 16 Bit, uncompressed .wav format).

In the further analyses, only the recordings of the study females that were housed together with an adult male and accepted sexual behaviour (head placing, mounting, copulation) from him during observation period were included (*N* = 8; Table 1). During the observations, the display of sexual behaviour was noted irrespective of the current focal animal. A sequence of consecutive days (±1) on which the female accepted the sexual behaviour was considered the female’s receptive period (*d0*) (see Jenikejew *et al.*, 2021 for a detailed description). Non-receptive periods were defined as 3 and 6 days (±1) before the first display of sexual behaviour as well as 3 and 6 days (±1) after the last day of sexual behaviour display.

Overall, 75 hours of data were analysed: 20 hours at Zoo Osnabrück (April 2014), 20 hours at Zoo Augsburg (July/August 2014), 5 hours at Serengeti-Park Hodenhagen (April/May 2015), 20 hours at Zoo Schwerin (April/May 2018) and 10 hours at Zoo Münster (July/August 2018).

### Vocal and behavioural analysis

Video recordings were synchronized with respective audio recordings and analysed using the *Observer XT* software (version 12, Noldus Information Technology, Netherlands; Noldus, 1991). The analysis was conducted by two different observers. The Cohen’s Kappa coefficient was determined among them by comparing 15 pilot observations (total of 100 minutes). κ values were ≥0.95, indicating a high interrater reliability (Landis and Koch, 1977).

Vocalizations were detected by auditory identification and categorized according to literature (Cinková and Policht, 2014; Linn *et al.*, 2018; Owen-Smith, 1973; Policht *et al.*, 2008). For each vocalization, the respective call type, sender as well as potential receiver were noted (see Jenikejew *et al.*, 2020 for a detailed description). Analysis focused on the contact call Pant and the agonistic call types Hiss and Grunt only, as a previous study demonstrated sex-specific differences in call rates, and hence a potentially relevant role in mating context for these call types (Jenikejew *et al.*, 2020). Pants are described as bouts of repetitive calls produced during inhalation and exhalation, mainly emitted in social contact and mating contexts (Policht *et al.*, 2008). In contrast, the broadband noisy nasal sound Hiss and the low-frequency growling vocalization Grunt are both uttered exclusively in aggressive contexts in order to displace or threaten the potential receiver (Policht *et al.*, 2008).

Behaviour was coded considering proximity measurements of the focal female to present group members, taking adult body length (2.5–3 m; Owen-Smith, 1973) as the unit of measurement. The duration each focal female spent in close proximity (≤ 1 body length) to each group member was noted.

For each focal female, the occurrence of affiliative, aggressive and defensive interactions and the respective interaction partner as well as olfactory behaviour were noted (see ethogram in Table 2). Affiliative interactions included social exploration of the interaction partner as well as socio-positive behaviour and presenting. Aggressive interactions were coded when the focal female displaced, attacked, chased, pushed or clashed horns, etc., with the interaction partner, whereas defensive interactions were coded when the focal female avoided or escaped from the interaction partner. Olfactory behaviour comprised marking as well as sniffing and flehming.

**Table 2:** Ethogram of olfactory, affiliative and agonistic behaviour of captive SWRs

	Behaviour	Description
Olfactory	Marking	Focal animal urinates intermittently or spreads its defecation with its hind legs
	Sniffing	Focal animal explores ground/objects or urine/faeces by inclining towards it, ‘sliding’ along the surface with the snout
	Flehming	Focal animal opens its mouth and curls back its upper lip exposing its upper gum while inhaling
Affiliative	Following	Focal animal moves after a conspecific while it changes the location
	Snout contact	Focal animal explores the body of another conspecific (except the snout) with its snout
	Social flehming	Focal animal flehms while scenting a defecating/urinating conspecific close by
	Naso-nasal sniffing	Focal animal contacts the nasal region of another conspecific with its own snout
	Ano-genital sniffing	Focal animal contacts the ano-genital region of another conspecific with its own snout
	Head placing	Focal animal lays its head on the back of another conspecific (only for bulls)
	Body contact	Focal animal touches or brushes another conspecific while moving with any part of its body (except snout) or rubs itself against a conspecific
	Presenting	Focal animal lifts up its tail while the bull is standing behind
	Mounting	Focal animal climbs with its forelegs on another conspecific (only for bulls)
	Copulation	The animals mate: the bull inserts his penis into the cow
Aggressive	Displace	Focal animal incites a conspecific to change its position/location after approaching or agonistic interaction
	Nodding	Focal animal swings its head back and forth
	Lifting	Focal animal lifts another conspecific’s head or leg with its head/horns
	Staring	Focal animal is standing horn to horn in front of another conspecific with an uplifted head
	Pushing	Focal animal presses any part of its body against another conspecific making it change the position/location
	Chasing	Focal animal follows another conspecific, which tries to keep the Focal animal at a distance, in a trotting manner
	Feigned attacking	Focal animal moves with a lowered head towards another conspecific and stops suddenly without causing body contact
	Attacking	Focal animal hits its horn against another conspecific
	Horn clashing	Escalated confrontation following attacking involving both animals hitting their horns against each other
Defensive	Avoiding	Focal animal changes its position or location after being approached by a conspecific, agonistic interaction with or agonistic vocalization from it
	Escaping	Focal animal moves away from a conspecific in a trotting manner after an agonistic interaction

### Faecal sample collection

Hormonal levels were measured non-invasively by analysing excreted hormone metabolites in the faeces. Individual faecal samples were collected on 2–7 days weekly over an average period of 32 days (Table 1). To ensure a clear individual assignment and to collect the samples at an approximately same time of the day, faecal samples were collected in the morning after the study animals were housed separately during the night (Brown *et al.*, 2001; Carlstead and Brown, 2005). When females were housed together with their calf, dung piles were distinguished based on the size of the faecal boli. In Zoo Schwerin faecal samples were collected immediately after defaecation, as the animals were not separated during the night. Accordingly, the general time lag between defaecation and sample collection did not exceed 12 hours. Immediately after collection, samples were frozen and stored at −20°C until further analysis.

### Hormone metabolite determination

After defrosting, a representative subsample of 0.5 g was weighed from each faecal sample. Subsequently, 4.5 ml of 90% methanol (MeOH) was added, followed by 30 minutes of shaking. The mixture was centrifuged for 15 minutes at 1000 × g (Rotanta 46RC, Hettich GmbH & co, Tuttlingen, Germany). An aliquot of 0.5 ml supernatant was then transferred to an Eppendorf vial and diluted 1:1 with distilled water to obtain a 45% MeOH extract. Of this final extract, 20 μl was used for each enzyme immunoassay (EIA) well. This sample extract was combined with 100 μl of enzyme label (1:2400 and 1:3200, for progesterone and oestrogens, respectively) and 100 μl antibody (1:6000 and 1:80.000, for progesterone and oestrogens, respectively). After overnight incubation at 6–8°C on a shaking platform (Ika Vibrax VXR, IKA® - Werke GmbH & co. KG, Staufen, Germany), the microtitre plates were washed four times (Hydrospeed, Tecan Group Ltd, Männedorf, Switzerland) before adding 150 μl substrate buffer (TMB) for 40 minutes at room temperature, without shaking. The enzymatic reaction was stopped by adding 50 μl 2 M sulphuric acid to each well and the absorbance was subsequently measured at 450 nm (Infinite M200, Tecan Group Ltd, Männedorf, Switzerland).

Faecal progesterone metabolites were quantified by an EIA according to Dehnhard *et al.* (2008) using a commercial progesterone antibody raised in rats (Sigma P1922) and a 4-pregnen-3,20-dione-3-CMO-peroxidase label. The cross-reactivities to other steroids were as follows: 4-pregnen-3,20-dione (progesterone), 100%; 5α-pregnan-3,20-dione (5α-DHP), 76.8%; 5α-pregnan-3ß-ol-20-one (5a), 5-pregnen-3ß-ol-20-one, 10.8%; 18.3%; <0.1% for 5ß-pregnan-3α-ol-20-one, 20α-dihydroprogesterone, pregnandiol, 17α-hydroxyprogesterone, testosterone, estradiol, and cortisol.

The inter-assay coefficient of variation (CV) (9 assays), based on a low-quality control (LQC) sample and high-quality control (HQC) sample, both fitting the linear range of the curve and run in duplicate, was 10.3% and 7.4%, respectively. The intra-assay CV, determined on two biological samples including low and high concentration respectively (16 repeats in duplicate each), was 4.1% and 4.2%, respectively. The range of the calibration curve (standard progesterone) was 0.2–100 pg/20 μl. The linear range, between B80 and B20, was determined between 1.80 and 20.00 pg/20 μl and refined to 6.25–20.00 pg/20 μl based on parallelism data after visual inspection confirmed with an overall CV of <20% between the different sample dilutions. All EIA measurements were performed in duplicate with acceptance criteria of a CV below 5%.

Faecal oestrogen metabolites were quantified by an EIA according to Carnaby *et al.* (2012) using a polyclonal antibody raised in rabbits to 1,3,5(10)-estratrien-3,17b-diol-17-HS-BSA and a 1,3,5(10)-estratrien-3,17b-diol-17-HS-peroxidase label. The cross reactivities to oestrogens were as follows: 1,3,5(10)-estratrien-3,17b-diol (17b-E2), 100%; 1,3,5(10)-estratrien-3,17-one (estrone), 100%; 1,3,5(10)- estratrien-3,17a-diol (17a-E2), 66%; 1,3,5(10)-estratrien- 3,16a,17b-triol (oestriol), 1.5%; and 0.1% for 19-nortestosterone, P4 and testosterone.

The inter-assay CV (7 assays), based on LQC and HQC samples, both fitting the linear range of the curve and run in duplicate, was 5.73% and 12.74%, respectively. The intra-assay CV, determined on two biological samples including low and high concentration respectively (16 repeats in duplicate each), was 3.46% and 2.97%, respectively. The range of the calibration curve (standard 17b-E2) was 0.2–100 pg/20 μl. The linear range, between B80 and B20, was determined between 0.95 and 15.11 pg/20 μl and refined to 0.95–3.12 pg/20 μl based on parallelism data after visual inspection confirmed with an overall CV of <20% between the different sample dilutions. All EIA measurements were performed in duplicate with acceptance criteria of a CV below 5%.

Progesterone metabolites were analysed in the faecal samples of all study females, while oestrogen metabolites were not analysed in the faecal samples of study females from Dortmund and Knuthenborg (Supplementary 1). Hormone metabolite values were dated with a delay of 1 day based on a previous study by Hindle and Hodges (1990) that described a peak of metabolite concentrations in faeces 24 hours after the intravenous injection of radiolabelled oestradiol-17β and progesterone in SWR females.

#### Definition of a hormonal cycle

A hormonal cycle was determined by assessing the dynamics of fPM levels indicating a periodic pattern. For each study female that was sampled on at least 3 days weekly (see Table 1), a baseline fPM value was determined using an iterative process previously established by Brown *et al.* (2001) in which the fPM values that exceeded $\overline{x}$ + 1.5 × SD were excluded. The new average values were recalculated, and the elimination process continued until no fPM values exceeded $\overline{x}$ + 1.5 × SD.

A cyclic pattern was identified if the fPM values remained around baseline level for at least two consecutive values and were preceded or followed by a peak above baseline by at least 50% that remained elevated for at least three consecutive values, eventually declining back to fPM levels around baseline level for at least two consecutive values (Brown *et al.*, 2001; Patton *et al.*, 1999). One-point peaks were ignored (Brown *et al.*, 2001).

### Data analysis

Statistical tests were derived from a previous study (Jenikejew *et al.*, 2021) and calculated in RStudio (version 4.0.2; R StudioTeam, 2016). The significance level was set at *P* ≤ 0.05. Normal distribution of individual hormone metabolite levels as well as mean hormone metabolite levels was verified using the Kolmogorov–Smirnov test and Q-Q plots calculated in SPSS (version 26; IBM Corp., 2019).

#### Differences across receptive and non-receptive periods

For the study females that were housed with adult bulls and were observed to show acceptance of sexual behaviour, signalling their receptivity (*N* = 8), the differences in fPM and fEM concentrations as well as behavioural and vocalization rates between receptive and non-receptive periods were investigated.

In order to assess the level of social cohesion between focal female and adult male, daily proximity rates were calculated for each one of the focal female–male dyads by dividing the duration the focal female spent in close proximity (≤ 1 body length) to the male by the total observation time of the focal female on that day. Daily proximity rates were indicated as a proportion ranging from 0 to 1. Thus, a value of 1 indicated that the female–male dyad spent the full observation time together, whereas a value of 0 indicated that the female–male dyad spent no time together. Daily interaction rates were calculated by dividing the number of (i) affiliative, (ii) aggressive and (iii) defensive interactions of the focal female with the male by the total observation time of the focal female on that day. Daily olfactory rates (marking, sniffing/flehming) were calculated by dividing the number of displayed behaviours by the total observation time of the focal female on that day. Daily directed call rates were calculated for each call type by dividing the number of calls the focal female uttered towards the male by the total duration the focal female spent in close proximity to the male on each observation day. Daily interaction, olfactory and call rates were indicated as number per hour.

Both daily fPM and fEM concentrations as well as daily behavioural and vocalization rates were determined as mean values across the receptive period (*d0*) and non-receptive periods (*d0+/−3*, *d0+/−6*). Subsequently, we investigated whether the different periods (*d0–6, d0–3, d0, d0 + 3, d0 + 6*) had an effect on the mean values by calculating linear mixed effect models (LMEs, ‘nlme’ package, ‘lme’ function) using the mean values of fPM levels, fEM levels, behavioural rates or vocalization rates as response variable and the periods as predictor variable, while controlling for ‘zoo’ and ‘ID’ as random factors and using a Gaussian distribution. Residuals were calculated for all LMEs (‘resid’ function) and subsequently verified for normality as well as for homogeneity of variances. A significant effect was determined by the likelihood ratio test (‘car’ package, ‘Anova’ function). Multiple comparisons between the receptive period and the non-receptive periods were Bonferroni–Holm adjusted (‘p.adjust’ function, ‘holm’ method).

As some behaviour and call types (marking, presenting, defensive interactions, Pant call rate, Grunt call rate) were only rarely observed, a statistical analysis was limited by zero inflation. For marking and presenting a chi-square test was calculated in SPSS with binomial data comparing the number of individuals, which displayed the behaviour during the different periods (*d0–6, d0–3, d0, d0 + 3, d0 + 6*). Defensive interactions as well as Pant and Grunt call rates were analysed descriptively, as they were displayed by a maximum of three different study females during a few periods.

#### Differences in baseline fPM concentrations

For the study females that were sampled at a sufficient rate to allow the detection of ovarian activity, non-pregnant baseline fPM levels were compared (*N* = 19).

We investigated if the occurrence of a hormonal cycle during the sampling period (yes/no) as well as the male state (acceptance of sexual behaviour from male/no acceptance of sexual behaviour from male/no direct contact to male) had an effect on the baseline fPM levels. For this purpose, we calculated an LME using the baseline fPM levels as response variable and the occurrence of hormonal cycle and male state as predictor variables. We controlled for ‘zoo’ as random factor and used a Gaussian distribution. The best fitting model (final model) was determined via backward stepwise elimination procedure (‘car’ package, ‘Anova’ function). To investigate significant effects of the main factor ‘male state’, a comparison across the three male states was conducted (‘ls means’ package, ‘lsmeans’ function, ‘Tukey’ adjustment for multiple comparisons). Residuals were calculated for LME (‘resid’ function) and subsequently verified for normality as well as for homogeneity of variances.

## Results

### Differences across receptive and non-receptive periods

#### Behaviour and vocalization

**Figure 1:**
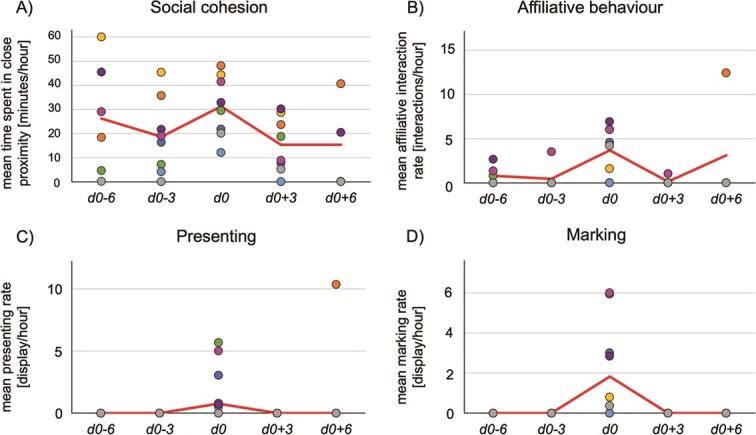
Behavioural rates of SWR females that were significantly affected by their receptivity. Mean values of (**A**) time spent in close proximity to the adult male, (**B**) affiliative interaction rate with adult male, (**C**) lifting up the tail when close to the adult male and (**D**) urinating intermittently. Each data point represents a study female during receptive (*d0*) and non-receptive (*d0* ± 3/6) periods. Red lines represent the mean values (A + B) or median values (C + D) over all females.

The period of female receptivity proved to have a significant effect on social cohesion between the study females and the adult males (ANOVA: χ2 = 17.500, df = 4, *P =* 0.002; Fig. 1A). Except during the non-receptive period *d0–6* (*P* = 0.097), study females spent significantly more time in close proximity to adult males during the receptive compared to the non-receptive periods (t ≥ |2.738|, 0.002 ≤ *P* ≤ 0.012, 0.009 *≤ P_corr_ ≤* 0.036).

**Figure 2:**
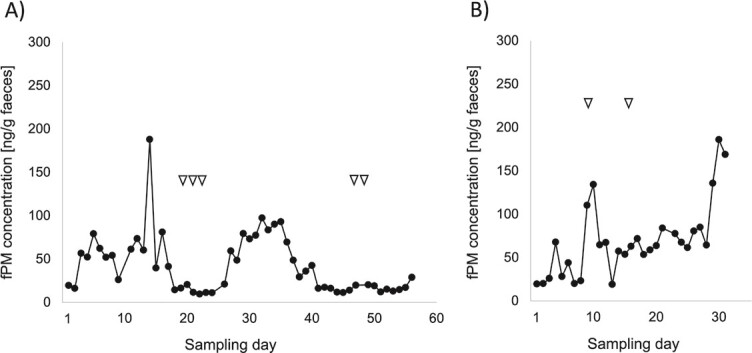
Concentrations of fPM in SWR females. Triangles represent acceptance of sexual behaviour from male, indicating receptivity. (**A**) Study female with regular oestrous cycle and coinciding receptivity. (**B**) Study female with an irregular oestrous cycle and sporadic display of receptivity.

Furthermore, the period of female receptivity proved to have a significant effect on female affiliative interactions towards males as well (ANOVA: χ2 = 13.605, df = 4, *P* = 0.009; Fig. 1B). The pairwise comparisons revealed that affiliative interaction rates were significantly higher during the receptive period *d0* compared to the non-receptive period *d0 + 3* (*t* = −2.786, *P* = 0.049, *P_corr_* = 0.043). During the non-receptive periods *d0–6* and *d0–3* affiliative interaction rates showed to be lower compared to the receptive period *d0*, though not significantly after the Bonferroni–Holm correction (0.011 ≤ *P* ≤ 0.049, 0.056 ≤ *P*_*c*orr_ ≤ 0.097). Focusing on the affiliative behaviour of presenting in particular demonstrated that with the exception of one female this behaviour was displayed exclusively during the receptive period (χ^2^ = 20.938, df = 4, *P <* 0.001; Fig. 1C).

No significant effect of receptive and non-receptive periods on aggressive interaction rates towards males could be revealed (*P =* 0.094).

Furthermore, only three study females displayed defensive interactions towards males overall. Two of them showed defensive behaviour only during one period (*d0* and *d0 + 3*, respectively), while the third female behaved defensively during *d0* and *d0+6*. The interaction rates were around the same level for all three females (1.232 ± 0.198 interactions/hour). Hence, no specific or pattern-like distribution of the defensive interaction rates over the receptive and non-receptive periods could be described.

No significant effect of female receptivity on sniffing and flehming rate could be found in the study females (*P* = 0.066). However, marking behaviour was significantly higher during the receptive period compared to the non-receptive periods before and after, as the females displayed this olfactory behaviour exclusively during their receptive period (χ^2^ = 24.440, df = 4, *P <* 0.001; Fig. 1D).

Even though female receptivity proved to have a significant effect on the Hiss call rate towards males (ANOVA: χ^2^ = 11.599, df = 4, *P* = 0.021), the further pairwise comparisons did not reveal any significant differences in Hiss call rate between the receptive period and the non-receptive period before and after (*P* ≥ 0.063, *P_corr_* ≥ 0.250). Similarly, no specific distribution of the other agonistic call type Grunt could be described during the receptive and non-receptive periods. The Grunt was uttered towards males by only two study females: One of them emitted the call only during *d0* at a rate of 3.750 calls/hour, while the other one emitted it during *d0* as well as during *d0+6*.

Regarding the Pant, again only two of eight study females emitted this call type at all. Interestingly, the ones that did utter Pants did so exclusively during the receptive period *d0,* though at different call rates of 1.050 calls/hour and 9.531 calls/hour.

#### Faecal hormone metabolites

No significant effect of female receptivity on mean fPM or fEM concentrations (*P* = 0.075 and *P* = 0.081, respectively) could be found in the study females that were housed with adult bulls and were observed accepting sexual behavioural from them. Accordingly, there were no significant differences in mean faecal hormone metabolites between the receptive and non-receptive periods before or after.

### Hormonal cycles

Overall, for 21 of the study females a sufficient number of faecal samples was collected, allowing the detection of ovarian activity. Six of these study females signalled their receptivity towards the bull, eight did not accept sexual behaviour from the bull and seven were housed without direct contact with a bull.

A hormonal cycle could be determined in seven females. While in these cases fPM levels showed a typical cyclic pattern of a clear nadir around baseline levels of ~1 week, framed by fPM levels that were more than 50% higher than the baseline levels, fEM concentrations did not show any specific patterns in their dynamics and fluctuated from day to day at approximately constant levels. For the other 14 females no hormonal cycle could be described during the sampling period, as both fPM and fEM levels were rather flat and fluctuated irregularly.

Of the six study females who indicated their receptivity by accepting sexual interactions from adult bulls, five were those who were identified to have had a hormonal cycle (example Chris; Fig. 2A). One of them, however, was found to have had an irregular fPM profile that did not indicate a hormonal cycle (Amalie; Fig. 2B).

**Figure 3:**
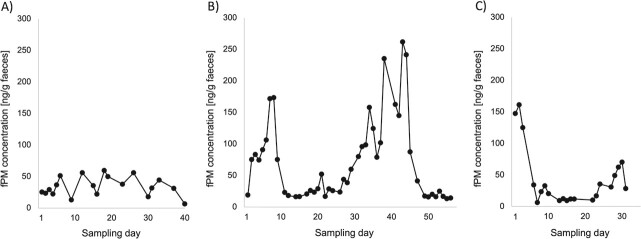
Concentrations of fPM in SWR females. (**A**) Study female without a regular oestrous cycle and no display of receptivity while housed with adult male. (**B**) Study female with a regular oestrous cycle but no display of receptivity while housed with adult male. (**C**) Study female with a regular oestrous cycle while housed separately from adult male.

**Figure 4:**
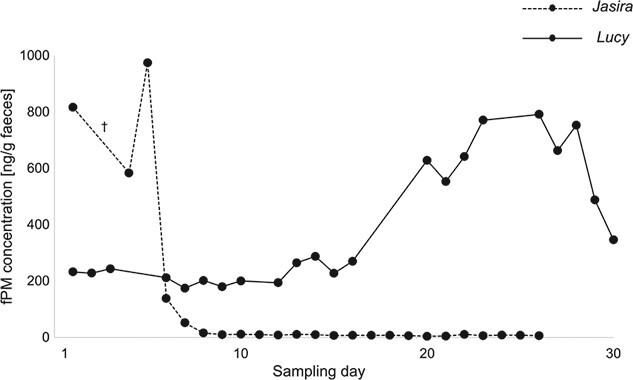
Concentrations of fPM in two pregnant SWR females. Jasira during late pregnancy, stillbirth occurred on third sampling day (†). Lucy during early pregnancy, mating occurred 2 months before first sampling day.

Of the eight study females that were kept with an adult male but did not indicate any receptivity, no hormonal cycle could be determined (example Temba; Fig. 3A), except for one female that displayed a clear periodical pattern in her fPM profile (Baby; Fig. 3B). In addition, the hormone profile of one of the females showed particularly high fPM values compared to the others (Lucy; Fig. 4): Approximately 2 weeks into sampling, her fPM levels increased by more than three times the baseline levels within 4 days and remained substantially elevated. It should be noted that this female mated 2 months before the beginning of the data collection and based on weekly blood sample analysis pregnancy was confirmed by the zoo veterinarian at the end of it.

Finally, of the seven study females that were housed separately from the adult males, a hormonal cycle could be identified in one (Tala; Fig. 3C). The hormone profile of another study female housed separated from an adult bull showed comparatively high fPM levels of about 800 ng/g faeces at the beginning of the sampling period that suddenly dropped to a one-hundredth of the maximal value and remained at a very low level of ~10 ng/g faeces (Jasira; Fig. 4). This female endured a stillbirth and the dead infant was delivered by the attending veterinarian 3 days into sampling, only a few days away from estimated parturition.

### Differences in baseline fPM concentrations

**Figure 5:**
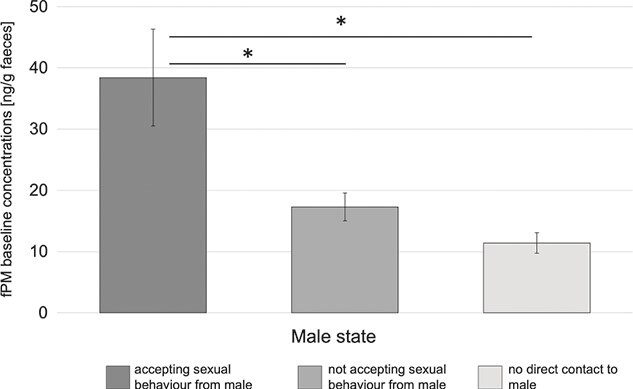
Mean and standard error of the fPM baseline concentrations in SWR females with different states of accepting male sexual behaviour; *N*_Accepting_ = 6, *N*_NotAccepting_ = 7, *N*_NoMale_ = 6. *P ≤ 0.05; pairwise comparison based on Tukey adjustment.

For the 19 non-pregnant study females that were sampled in a sufficient quantity, no significant effect of the hormonal cycle on the baseline fPM concentrations could be detected (*P* = 0.281). Hence, there was no difference in baseline fPM levels between females with a hormonal cycle during the sampling period and the ones without. In contrast, ‘male state’ turned out to have a significant effect on the fPM baseline concentrations (ANOVA: χ2 = 18.875, df = 2, *P* < 0.001). A subsequent comparison revealed that study females that accepted sexual behaviour from males had significantly higher fPM baseline concentrations compared to study females that did not accept sexual behaviour from males (df = 7, *t* = 3.145, *P* = 0.038) or did not have any direct contact with males at all (df = 8, *t* = 3.793, *P* = 0.013; Fig. 5).

## Discussion

The findings of the present study provide comprehensive insights into the interplay of behaviour, vocalization and hormonal states for SWR females. Their receptive period, indicated by accepting male sexual behaviour, clearly coincided with distinct peaks in female affiliative and olfactory behaviour. Furthermore, by analysing the profiles of fPM concentrations, we were able to identify different reproductive states and demonstrate a coincidence between observed receptivity and hormonal oestrus for the majority (five of six) of the females. In these females, the passive acceptance of male sexual interactions as well as the active behavioural signalling of their readiness to mate were observed during low fPM concentrations, on average between 5 and 11 days before the fPM levels began to rise again, indicating the beginning of the luteal phase.

While female affiliative behaviour towards males as well as their rate of social cohesion with males generally peaked during receptivity, olfactory-related behaviour such as presenting and marking stood out specifically, as females displayed those almost exclusively during the time they accepted sexual interactions from males. In addition, Pant calls were uttered, even if only by very few females, exclusively during their receptive period, too. Hence, these findings not only provide the first statistical evidence for female behavioural indicators of their receptivity described in previous studies (e.g. Radcliffe *et al.*, 1997), but they also prove that olfactory communication is particularly crucial for signalling their reproductive state.

When comparing female behavioural and vocal indicators of receptivity with the ones of males described in a previous study (Jenikejew *et al.*, 2021), it becomes obvious that while there are some similarities with regards to seeking vicinity to the other sex and exchanging socio-positive interactions, there is a difference in focus of signalling the willingness to mate. While males primarily seem to indicate their interest and desire for mating via vocal communication, females seem to signal their readiness for mating via olfactory communication by marking or lifting up their tail, and in doing so, providing the opportunity for the males to absorb essential olfactory cues.

Hence, the different characteristics of behavioural changes in the reproductive context in females and males described in the present and previous studies (Jenikejew *et al.*, 2021) complement each other and provide a complete picture of the courtship and mating ritual in captive WRs. During their receptive period, females offer the males more opportunities to take in their olfactory cues by increasingly spraying urine and lifting up their tail. As soon as the males have absorbed the respective stimuli, they seek out the female and signal their interest by uttering the Pant call while approaching. These behavioural patterns correspond to the social organization and spatial distribution of the animals in the wild (Owen-Smith, 1975; Owen-Smith, 1988; Shrader and Owen-Smith, 2002) and, interestingly, seem to be maintained in captive conditions as well.

In contrast, the female aggressive behaviour and the agonistic call types Hiss and Grunt towards the males did not change throughout the receptive and non-receptive periods. Even though we would have expected the aversive behaviour of the females to decrease during their receptive period so they would act less rejecting towards the bulls, it turned out that the rates of agonistic behaviour and call types Hiss and Grunt remained unchanged during receptivity and thus, only the affiliative and olfactory behaviour increased.

Regarding the development of fPM and fEM levels, no significant differences between the receptive period and the non-receptive period were found in this study, contrasting to behavioural rates. We would have expected a peak of fEM levels during the receptive phase or shortly before, indicating the imminent ovulation, but we could not confirm this. A possible explanation could be that fEM measures in WR have so far failed to create conclusive data and hence, to provide a useful assessment tool for the follicular activity, as previously stated by several studies (Brown, 2018; Brown *et al.*, 2001; Roth *et al.*, 2018). One reason for fEM measures not being a conclusive indicator might the possibility of oestrogen metabolites being primarily excreted in urine instead of faeces (Brown, 2018). However, as shown in the radio metabolism study by Hindle and Hodges (1990), in WR oestrogen metabolites were predominantly found in faeces. Another explanation is given by Schwarzenberger and Brown (2013), according to which fEM measures being little informative is mainly attributable to the low quantities of oestrogens produced by the follicles in African rhinoceros species, as studies on Indian rhinoceros (*Rhinoceros unicornis*) females, having particularly large follicles, proved that faecal oestrogen measures in this species can be implemented to assess the cycle phase (Schwarzenberger *et al.*, 2000; Stoops *et al.*, 2004). Nevertheless, we expected that a higher sampling rate may have improved the accuracy of the assessment and indicated the volatile peak in oestrogen levels during the follicular phase. However, even sampling at a daily rate as in three of our oestrous study females did not result in any distinct oestrogen pattern that could have signalled a hormonal oestrus. Therefore, fEM profiles could not be used for the identification of ovarian activity in SWR females.

As for the fPM levels, the lack of differences in concentrations between the receptive and the non-receptive period falls in line with the nadir of progesterone levels described for the follicular phase. In WR females, the increase in progesterone concentrations has been previously estimated to begin between 6 and 9 days post-ovulation (Patton *et al.*, 1999; Radcliffe *et al.*, 1997; Schwarzenberger *et al.*, 1998). Hence, it is likely that we would have detected increasing progesterone levels if we have included a longer non-receptive period into our analysis. However, for half of the study females that signalled their receptivity, the subsequent observation period and thus the sampling period lasted less than a week. This means that for future studies a longer observation period following the female oestrus should be targeted to facilitate a comprehensive characterization of the complete cycle. Nevertheless, these findings also provide additional support for the fact that there might be other hormonal messengers that elicit the behavioural changes during their receptive period in females.

By analysing the development of fPM concentrations throughout the observation periods, we were able to assign the hormonal profiles of 21 study females to hormonal patterns reflecting different reproductive states, e.g. cycling, non-cycling and pregnant. When comparing the baseline fPM levels between cycling and non-cycling females, we could not find any significant differences, suggesting that judging by this parameter alone is not sufficient for the identification of females that potentially cycle on a regular basis. These findings clarify that the comparison of individual hormonal values does not allow a direct classification of the reproductive state. Due to the limited sampling periods, it was not possible to determine if the females that did not cycle during that period were completely acyclic or had an irregular cycle. Moreover, for the cycling females we were not able to provide further information about the cycle type, since WR females are known to have extended cycles, in which the luteal phase of the ‘long’ cycles can last more than twice as long as the one in the 1-month ‘short’ cycles (e.g. Brown *et al.*, 2001; Patton *et al.*, 1999; Schwarzenberger *et al.*, 1999). Therefore, in order to characterize the non-cyclic and cyclic hormonal patterns in more detail, an endocrine monitoring over several months is necessary (Schwarzenberger *et al.*, 1999).

In five of the eight study females that signalled their receptivity by accepting sexual interactions directed towards them by adult bulls during our observation period, a hormonal cycle could be detected. In these five females, the receptive period temporally coincided with the nadir of the fPM levels and hence, with the follicular phase. This concurrence provides clear endocrine evidence that the behavioural indicators described above do in fact mark the fertile phase of the SWR females. For two of the other females the sampling rate was not sufficient in order to reliably detect a hormonal cycle (Claudia and Marsita).

One female (Amalie), however, presented a noteworthy exception to the coincidence between the receptive period and the follicular phase. Even though this female clearly accepted sexual behaviour from the bull, her fPM profile did not reveal the expected cyclic pattern, as it was lacking a nadir that should have lasted over several consecutive values and showed rather variable excretion instead. Therefore, it was not in accordance with what has been described as the definition of a hormonal cycle in WRs by previous studies (Brown *et al.*, 2001; Patton *et al.*, 1999). This irregularity in ovarian activity was also reflected in the expression of the behavioural patterns that indicated her receptivity. While in the other study females the receptive phase occurred accumulated over the course of several consecutive days, the female in question displayed the behavioural indicators rather sporadically with several days between the occasions. Moreover, Amalie displayed particularly high rates of presenting that, however, did not coincide with her receptive period, meaning that although she accepted sexual behaviour from the bull, her active signalling of receptivity did not concur with this period. Hence, we would need to carefully reconsider if the displayed acceptance of sexual behaviour truly represented a receptive period in this female, as it was irregular and did not correspond to the normal case observed in this study. It remains to be noted that due to the lack of available information we cannot provide a possible explanation for the described irregularity, e.g. ovarian cysts or other reproductive pathologies (e.g. Hermes *et al.*, 2004, 2006). What can be ruled out, however, is early pregnancy, since the bull was not able to fully penetrate the females due to a previous penis fracture. Furthermore, it is known that Amalie mated successfully with a new bull ~5 years after data collection, but endured a stillbirth shortly before parturition.

On the one hand, this exceptional case demonstrates that the inconsistency between endocrine state and behavioural patterns can be used as a potential indicator for aberrations in the reproductive cycle of the females. On the other hand, it also gives rise to the question of whether there might be another driver for the behavioural changes in females than the metabolites analysed in this study. Findings from another study female (Baby) point into the same direction, as we could describe a hormonal oestrus for her while she did not signal her receptivity towards the male. In view of the fact that she was one of the oldest study females it might be concluded that while aged females might still experience an endocrine cycle, they do not necessarily display the respective behaviour and hence are not considered being reproductively active anymore. Therefore, further studies that would identify the potential hormone driver of behavioural changes during oestrus as well as characterize its function and development in different reproductive stages would be necessary.

The example of a study female (Tala) with a hormonal oestrus but no access to an adult bull provides clear evidence that WR females are spontaneous ovulators, as already described by Roth (2006). The fact that at the time of the sampling period Tala also had a one-and-a-half-year old calf with her and still cycled proves that her postpartum acyclicity already ended, which falls in line with previous studies that reported WR females to resume cycling about 6–12 months after parturition (Hermes *et al.*, 2004; Owen-Smith, 1975; van der Goot *et al.*, 2013). Nevertheless, male stimulation still appears to be required in order to show the full female behavioural repertoire that signals receptivity, as study females with direct contact to bulls did not only accept their sexual interactions but also displayed other behavioural and vocal indicators, such as presenting, marking and panting, which were completely absent in Tala. Similar links between male stimuli and female sexual behaviour have been shown in other mammalian species that are spontaneous ovulators. In rats (McIntosh *et al.*, 1978) and mice (Hammerschmidt *et al.*, 2009) male ultrasonic vocalizations elicited approaching and solicitation behaviour in the females, while in sheeps and goats (reviewed by Delgadillo *et al.*, 2009) as well as in many primate species (reviewed by Rooker and Gavrilets, 2020) the mere introduction of a male induces receptive behaviour in the females. However, this type of behavioural change in females, triggered by the absence or presence of male stimuli, has not yet been systematically studied in WR. So far there are only individual reports about the introduction of a new bull stimulating female’s cycle in captivity as well as in the wild (Patton *et al.*, 1999; van der Goot *et al.*, 2015). Our study provides additional evidence for a potential male effect in SWR, as the male state but not the presence of hormonal cycle proved to have an effect on baseline fPM concentrations that were significantly higher in the study females that were sexually active and accepted the courtship behaviour of the males compared to those who had contact with males, but did not show sexual activity or had no direct contact with males at all. This would suggest that not hormonal cyclicity itself, but direct interaction with a male is associated with higher fPM levels.

In spite of these intriguing findings, conclusions for breeding management should be treated with caution, especially in view of the study’s rather small sample sizes and the large scattering of the data. Therefore, a reliable comparison of fPM baseline values, e.g. in order to determine the reproductive state of SWR females in different zoos, would not yet be possible and requires further research that systematically focuses on the male effect in WR.

Finally, the fPM profiles of the pregnant study females proved to correspond with previous descriptions of hormonal profiles in pregnant WR, describing elevated faecal pregnane levels with a steep increase exceeding luteal concentrations occurring around 3 months post-conception and remaining at high levels up until shortly before parturition (Hildebrandt *et al.*, 2007; Patton *et al.*, 1999; Pennington *et al.*, 2020; van der Goot *et al.*, 2013). One of the two pregnant females endured stillbirth during late pregnancy, while the other one was at the early stage of pregnancy. Reportedly, the latter one gave birth to a female calf in November 2019, which corresponds the estimation of her being 2–3 months pregnant during our observation period in September/October 2018. We do not have further information on what maternal or embryonic factors might have caused the embryo loss in the first female. Overall, these particular fPM profiles not only provide an adequate illustration of fPM profiles in pregnant SWR females but also lend strong support for the biological validation and reliability of the hormone assay implemented in this study.

In conclusion, our results provide a comprehensive list of the behavioural indicators that female SWR use in order to signal their receptivity. Thereby, it is not only the passive behavioural patterns such as allowing a male to approach and ultimately accepting sexual interactions that indicate the receptive phase, but also active behaviour that is specifically related to olfactory communication including presenting and marking. Furthermore, we confirmed that fPM but not fEM levels are suitable for describing ovarian activity in SWR females. Using the profiles of fPM concentrations that are characteristic for an ovarian cycle, we were able to identify different reproductive states of the females during the sampling period: pregnant, cycling and non-cycling. For the majority (five of six) of study females that indicated their receptivity by accepting sexual behaviour from bulls, the receptive period coincided with hormonal oestrus.

Combining the present results on the females’ endocrine state, behaviour and vocalization throughout their oestrus with the complementary study on the males’ side (Jenikejew *et al.*, 2021) established behavioural and vocal indicators in both sexes that create an intuitive and substantial basis for the reproductive monitoring of captive SWR on a daily basis. We found that there does both exist a female effect on the testosterone metabolite levels of the males (Jenikejew *et al.*, 2021) and a male effect on the progesterone metabolite levels of the females. In addition, we were able to show that physical contact is essential in order to elicit adequate behavioural and vocal indicators of female receptivity in both sexes. With regards to practical implementations of these behavioural insights for the captive management of WR, it would be necessary to create auditory and olfactory access between males and females. However, the present as well as previous studies (Jenikejew *et al.*, 2020; Jenikejew *et al.*, 2021) showed that females generally behave in a rather rejecting manner towards males and, except for the receptive period, actively keep them at a distance using aggressive call types such as Hiss and Grunt, which are often accompanied by behaviours such as displacing or attacking. Therefore, housing the whole group together at all times might either lead to increased stress or, quite the contrary, unintentional habituation that would result in behavioural changes crucial for mating, in that they would not be displayed intensively enough to attract the mating partner (Lindburg and Fitch-Snyder, 1994). This kind of sibling-like relationship has been especially observed in WR that were kept solely as a breeding pair, which often led to a silent oestrus in the female (Schwarzenberger *et al.*, 1999). Conclusively, simulating natural conditions by temporarily separating the adult bulls from the group, but creating regular possibilities for the breeding pair to exchange olfactory and acoustic cues might be a worthwhile approach to improve the reproduction rate in captive WR. Further studies could determine if olfactory cues from receptive females or acoustic cues from adult bulls might trigger the same behavioural and vocal responses in the opposite sex as physical presence. Ultimately, these findings could be applied to monitor the males’ and females’ readiness to mate and thus, to coordinate the contact between the breeding pair.

## Funding

This work was supported by the Serengeti-Park Stiftung and the Deutsche Forschungsgemeinschaft (SCHE 1927/2-1).

## Author contributions statement

J.J. collected part of the data, performed part of the video analysis, analysed the data, wrote the manuscript and prepared all figures. J.W. supervised the endocrine analysis and wrote the ‘Hormone metabolite determination’ section. M.D. supervised the endocrine analysis. M.S. provided the grant and designed and supervised the study. All authors contributed to and reviewed the manuscript.

## Data availability statement

Data used for the manuscript are included in the supplementary information. Video and audio data are stored at the Institute of Zoology, University of Veterinary Medicine Hannover, Germany, and are available on reasonable request.

## Supplementary Material

Supplementary_Information_Jenikejew_et_al_2021_coab098
