# Supplementary material for: Linking socio-sexual and vocal behaviour with faecal progesterone and oestrogen metabolite levels in Southern white rhinoceros females
Source: Conserv Physiol. 2021 Dec 29;9(1):coab098. doi: 10.1093/conphys/coab098 (PMC8715736; doi:10.1093/conphys/coab098)
Supplement: Supplementary_Information_Jenikejew_et_al_2021_coab098 [file supplementary_information_jenikejew_et_al_2021_coab098.docx]

# Supplementary Information

**Supplementary 1:** Hormone parameters of faecal progesterone metabolites (fPM) and faecal oestrogen metabolites (fEM) in Southern white rhinoceros females. SD = standard deviation.

| **ID** | **Zoo** | **Mean fPM [ng/g]** | **Minimum fPM [ng/g]** | **Maximum fPM [ng/g]** | **SD fPM [ng/g]** | **Baseline fPM [ng/g]** | **Mean fEM [ng/g]** | **Minimum fEM [ng/g]** | **Maximum fEM [ng/g]** | **SD fEM [ng/g]** |
| --- | --- | --- | --- | --- | --- | --- | --- | --- | --- | --- |
| Amelie | Osnabrück | 69.18 | 19.16 | 185.73 | 42.15 | 53.64 | 7.40 | 2.95 | 14.63 | 2.63 |
| Marsita | Osnabrück | 74.83 | 23.07 | 132.93 | 29.67 | 64.05 | 11.44 | 8.98 | 17.00 | 2.56 |
| Lia | Osnabrück | 33.42 | 5.02 | 60.37 | 21.72 | 33.42 | 7.07 | 4.94 | 12.35 | 2.19 |
| Chris | Augsburg | 42.58 | 9.72 | 187.92 | 33.72 | 13.91 | 6.45 | 3.66 | 18.12 | 2.21 |
| Kibibi | Augsburg | 75.77 | 8.21 | 384.15 | 71.47 | 51.43 | 5.23 | 1.56 | 11.47 | 2.19 |
| Baby | Augsburg | 71.94 | 13.00 | 261.90 | 65.85 | 16.06 | 7.01 | 4.21 | 16.78 | 2.27 |
| Shakina | Dortmund | 7.20 | 2.88 | 13.36 | 2.93 | 5.10 | - | - | - | - |
| Jasira * | Dortmund | 113.72 | 4.32 | 975.00 | 269.55 | 6.97 | - | - | - | - |
| Natala | Dortmund | 11.62 | 4.29 | 18.98 | 4.00 | 10.29 | - | - | - | - |
| Temba | Erfurt | 34.19 | 6.545 | 59.57 | 15.41 | 25.56 | 11.51 | 6.84 | 24.37 | 3.68 |
| Numbi | Erfurt | 29.59 | 8.49 | 63.42 | 14.35 | 20.54 | 7.12 | 5.39 | 8.62 | 1.00 |
| Uzuri | Hodenhagen | 69.62 | 17.05 | 165.11 | 53.72 | 55.98 | 5.33 | 3.69 | 6.89 | 1.18 |
| Kianga | Hodenhagen | 56.05 | 16.02 | 146.23 | 43.78 | 29.80 | 5.16 | 4.04 | 7.41 | 1.18 |
| Claudia | Hodenhagen | 58.73 | 16.50 | 123.90 | 48.38 | 58.73 | 6.12 | 4.89 | 8.29 | 1.25 |
| Doris | Hodenhagen | 20.86 | 2.94 | 56.93 | 14.15 | 15.32 | 9.50 | 6.80 | 16.86 | 2.43 |
| Cera | Gelsenkirchen | 12.25 | 8.47 | 17.61 | 2.40 | 11.87 | 4.97 | 3.20 | 7.67 | 1.19 |
| Tamu | Gelsenkirchen | 12.45 | 7.60 | 17.58 | 3.17 | 10.27 | 5.79 | 1.89 | 9.83 | 2.44 |
| Clara | Schwerin | 55.05 | 17.76 | 102.72 | 33.47 | 55.05 | 11.10 | 6.29 | 20.89 | 4.07 |
| Karen | Schwerin | 46.88 | 18.19 | 96.3 | 22.71 | 42.12 | 11.20 | 8.09 | 15.71 | 2.01 |
| Jane | Münster | 37.25 | 10.32 | 91.64 | 28.62 | 14.24 | 6.77 | 3.41 | 12.43 | 1.99 |
| Vicky | Münster | 12.41 | 9.351 | 14.907 | 1.79 | 12.41 | 8.94 | 6.83 | 11.36 | 1.79 |
| Yoruba | Amnéville | 20.87 | 9.35 | 47.93 | 9.14 | 16.50 | 15.14 | 9.78 | 20.89 | 3.41 |
| Hekaw | Amnéville | 17.79 | 7.85 | 44.7 | 10.09 | 11.26 | 13.12 | 7.22 | 19.34 | 2.72 |
| Lucy * | Amnéville | 389.05 | 175.35 | 791.4 | 222.60 | 209.88 | 17.74 | 12.62 | 21.92 | 2.74 |
| Tala | Amnéville | 43.21 | 6.24 | 161.1 | 46.13 | 10.10 | 11.35 | 5.16 | 15.74 | 2.34 |
| Jamala | Knuthenborg | 48.39 | 7.02 | 105.18 | 29.22 | 23.73 | - | - | - | - |
| Bodil | Knuthenborg | 35.41 | 4.65 | 270.54 | 48.49 | 13.05 | - | - | - | - |

* Study female was pregnant during sampling period
